# Survey on the extubation procedure in intensive care units in Buenos Aires, Argentina

**DOI:** 10.5935/0103-507X.20190027

**Published:** 2019

**Authors:** Mauro Federico Andreu, Marco Bezzi, Paula Pedace, Mariana Fredes, Iris Salvati, Andrés Leoz, Mariana Aguirre

**Affiliations:** 1 Universidad Nacional de la Matanza - Provincia de Buenos Aires, Argentina.; 2 Hospital Donación Francisco Santojanni - Ciudad Autónoma de Buenos Aires, Argentina.

**Keywords:** Airway extubation/adverse effects, Intermittent positive-pressure ventilation, Survey and questionnaires, Extubación traqueal/efectos adversos, Ventilación con presión positiva intermitente, Encuestas y cuestionarios

## Abstract

**Objective:**

To examine the usual practice of airway management during the extubation procedure through an online survey to professionals working in intensive care units in the Autonomous City of Buenos Aires and in the Province of Buenos Aires, Argentina.

**Methods:**

A cross-sectional descriptive study online survey was conducted from February 11 to March 11, 2013. A database was generated, and a voluntary and anonymous invitation to access the survey was sent by email to 500 participants.

**Results:**

Out of a total of 500 participants, 217 (44%) responded to the survey, of whom 59.4% were physical therapists. One hundred ninety-five (89.9%) professionals were working in adult care. Regarding the cuff deflation procedure and extubation, 203 (93.5%) performe endotracheal suctioning, and 27 (12.5%) use positive pressure. Approximately 53.5% of participants reported having experienced immediate complications with this procedure in the last three months. In all, 163 complications were reported, and stridor was the most prevalent (52.7%).

**Conclusion:**

Most professionals working in intensive care units in the Autonomous City of Buenos Aires and in the Province of Buenos Aires, Argentina, use endotracheal suctioning without applying positive pressure during extubation.

## Abstract (es)

**Objetivo:**

Conocer la práctica habitual del manejo de la vía aérea durante el procedimiento de extubación mediante una encuesta online a profesionales de las unidades de cuidados intensivos de la Ciudad Autónoma de Buenos Aires y Provincia de Buenos, Argentina.

**Métodos:**

Estudio descriptivo transversal de tipo encuesta online del 11 de febrero al 11 de marzo de 2013. Se envió por correo electrónico una invitación voluntaria y anónima para acceder a la encuesta a 500 participantes a partir de una base de datos confeccionada por los investigadores de este estudio.

**Resultados:**

De un total de 500 participantes, 217 (44%) respondieron la encuesta. El 59,4% son kinesiólogos. Ciento noventa y cinco (89,9%) profesionales se desempeñan en atención de adultos. Con respecto al procedimiento de desinflado del balón y extubación, 203 (93,5%) realizan aspiración endotraqueal y 27 (12,5%) emplean presión positiva. El 53,5% de los participantes informó haber tenido en los últimos 3 meses complicaciones inmediatas a este procedimiento. Se informaron un total de 163 complicaciones, siendo el estridor el más prevalente (52,7%).

**Conclusión:**

La mayoría de los profesionales de unidades de cuidados intensivos de la Ciudad Autónoma de Buenos Aires y Provincia de Buenos Aires, Argentina, emplea aspiración endotraqueal sin aplicar presión positiva durante el procedimiento de extubación.

## INTRODUCTION

Weaning from mechanical ventilation has not been well determined. There is no consensus on in its definition and practice, making it difficult to conduct and interpret epidemiological studies.^(1)^ In 2007, an International Consensus Conference on weaning from mechanical ventilation proposed a classification into three different groups (ICC groups) according to the quantity, duration, and results of spontaneous breathing tests and the extubation results.^(2)^

Extubation is the removal of the endotracheal tube when no longer required.^(3)^ It is a procedure frequently performed in intensive care units (ICU). However, this maneuver has received relatively little attention in the literature.^(4)^

During invasive mechanical ventilation, secretions accumulate in the subglottic space above the endotracheal tube (ETT) cuff. There is a risk that these secretions might pass into the distal airway during cuff deflation and extubation.^(5,6)^ To prevent this passage, the literature suggests several procedures: endotracheal suctioning (ES) and positive pressure (PP), either with a self-inflating bag or by using a ventilator.^(4,7-9)^

In laboratory studies, there was less aspirated volume with the use of PP during extubation.^(5,8)^ Hodd et al., showed there existed less aspiration using continuous positive airway pressure.^(5)^ Other authors observed less aspirated volume by combining pressure support and positive end expiratory pressure on the ventilator.^(8)^ However, according to a survey conducted in the United Kingdom, only 1.3% consider the use of PP during the procedure. On the other hand, 86.5% apply ES during cuff deflation and removal of the ETT.^(6)^

In our area, there is no information on how to perform extubation or the maneuvers commonly used during the procedure. Therefore, we conducted a survey directed to professionals working in ICUs in the Autonomous City of Buenos Aires and in the Province of Buenos Aires to identify the usual practice of airway management during extubation.

## METHODS

A cross-sectional descriptive study based on an online survey was conducted from February 11 to March 11, 2013. The study was approved by the Ethics and Research Committee of Hospital Santojanni. Doctors and physical therapists working in ICUs in the Autonomous City of Buenos Aires and in the Province of Buenos Aires, Argentina, were included. Through a non-probabilistic convenience sampling, participants were invited through a database generated for this purpose by the authors of the present study. Each participant was sent an invitation email containing a brief summary of the study and a link to access the survey online through the *Survey Monkey*^®^ tool. Because of the voluntary and anonymous nature of participation, informed consent was not required. Previously, a pilot test was conducted with 20 respondents to evaluate understanding, difficulty, and the time required to answer it.

**Figure 1:**
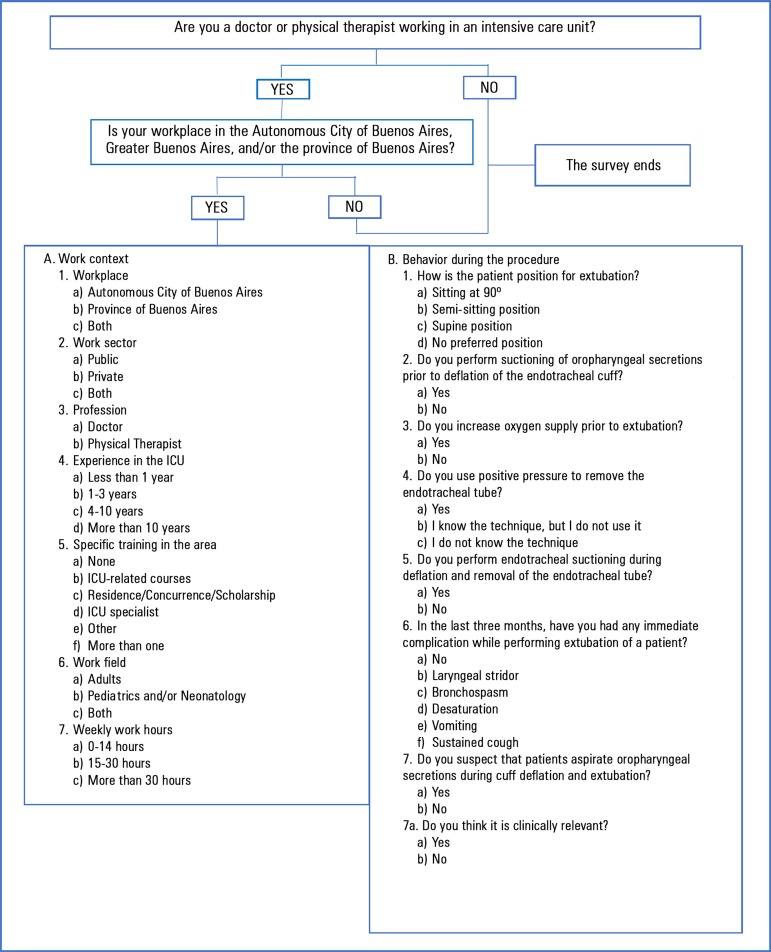
Survey administered to participants using the SurveyMonkey^®^ tool. ICU - intensive care unit.

The survey consisted of 15 short questions with simple answers: seven about the characteristics of the work context, eight aimed at exploring both the behaviors during extubation and the associated complications perceived by respondents in the last three months (Figure 1). Categorical variables were reported with as absolute numbers and percentages. The statistical software SPSS version 24 (SPSS Inc., Chicago, IL) was used for data analysis.

## RESULTS

Out of the 500 professionals who received the survey by email, 220 (44%) answered it. Three surveys were eliminated because they were incomplete, resulting in a total of 217 surveys analyzed. The characteristics of the respondents are reported in table 1.

**Table 1:** Characteristics of the respondents

Variables	N (%)
Workplace	
Autonomous City of Buenos Aires	124 (57.1)
Province of Buenos Aires	76 (35.1)
Both	17 (7.8)
Work sector	
Public	104 (47.9)
Private	68 (31.3)
Both	45 (20.8)
Experience in an ICU	
Less than 1 year	4 (1.8)
1 - 3 years	44 (20.3)
4 - 10 years	116 (53.5)
More than 10 years	53 (24.4)
Profession	
Doctor	88 (40.6)
Physical therapist	129 (59.4)
Specific training in the field	
None	2 (0.9)
ICU-related courses	14 (6.5)
Residence/Concurrence/Scholarship	39 (18)
Specialist in Intensive Therapy	81 (37.3)
Other	5 (2.3)
More than one	76 (35)
Work field	
Adults	172 (79.3)
Pediatrics and/or Neonatology	22 (10.1)
Both	23 (10.6)
Weekly work hours	
0 - 14 hours	18 (8.3)
15 - 30 hours	66 (30.4)
Over 30 hours	133 (61.3)

ICU - intensive care unit.

Out of the 217 professionals who responded to the survey, 81 (37.3%) perform extubation with the patient sitting at 90º, 124 (57.1%) with the patient in a semi-sitting position, and one (0.5%) in the supine position. Eleven did not report a preferred position (5.1%).

The aspiration of oropharyngeal secretions prior to deflation of the endotracheal cuff is performed by 214 (98.6%) professionals.

Seventy-seven (35.5%) respondents increase oxygen supply before extubation.

**Figure 2:**
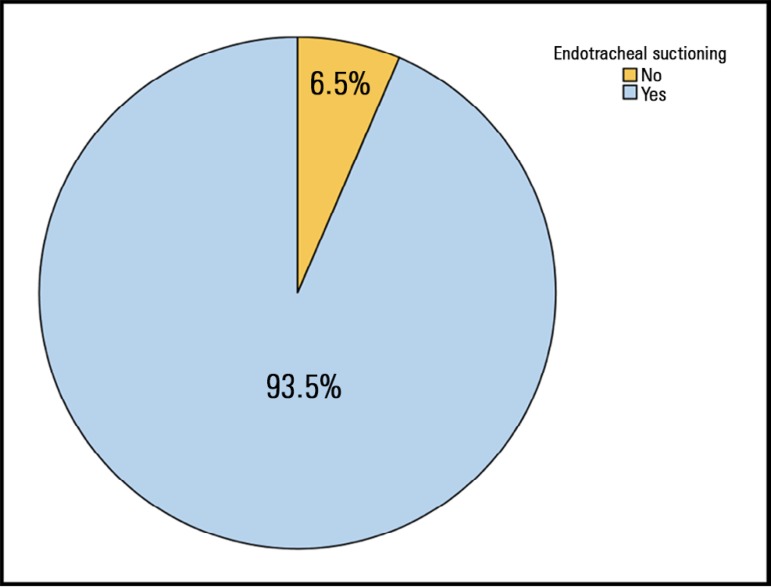
Use of endotracheal suctioning during cuff deflation and removal of the endotracheal tube.

**Figure 3:**
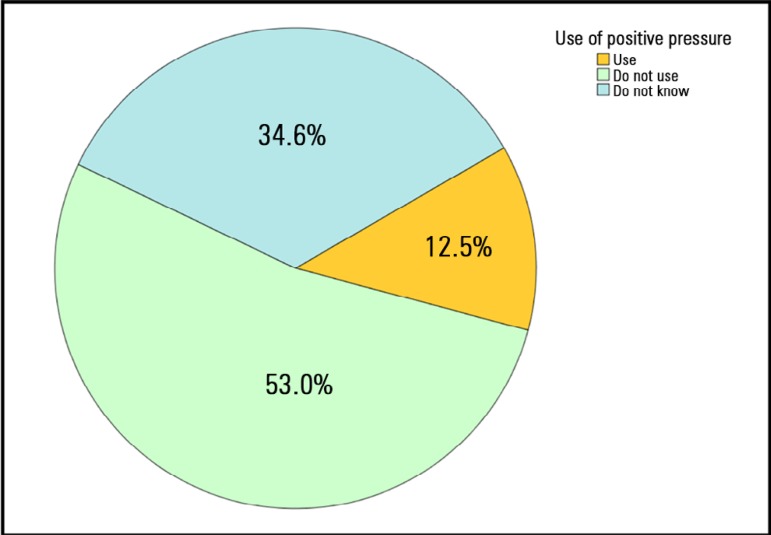
Use of positive pressure during cuff deflation and removal of the endotracheal tube.

Figures 2 and 3 show the frequency of using ES and PP during extubation, respectively. Out of those using PP during extubation (n = 27), 21 (77.8%) use a ventilator, and 6 (22.8%) use a self-inflating bag.

**Figure 4:**
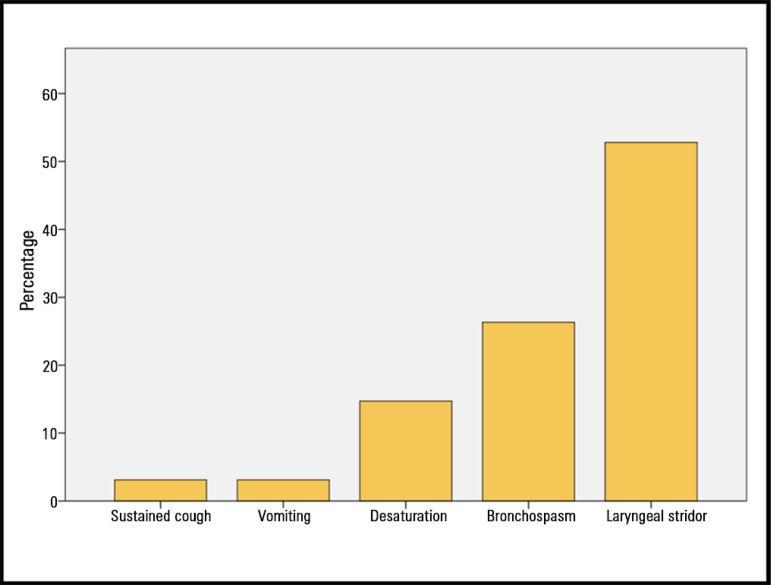
Immediate complications during extubation reported by respondents in the last three months.

Regarding the immediate complications of extubation in the last three months, 116 (53.4%) participants reported having experienced at least one. A total of 163 complications were reported, and stridor was the most prevalent (52.7%) (Figure 4).

One hundred fifty-four (71%) respondents answered they suspect that patients aspirate oropharyngeal material during cuff deflation and extubation. Out of these percentage, 59 (38.3%) thought that this aspiration is not clinically important.

## DISCUSSION

The main finding of the present study is that most professionals surveyed perform ES without using PP during extubation.

Out of all respondents, 93.5% use ES during extubation. Similar results were reported in another survey conducted in 179 ICUs in the United Kingdom, where 85% reported performing extubation with ES.^(6)^ According to the literature, the aim of using ES is to aspirate secretions passing into the airways by deflating the cuff, thus preventing their passage to more distal regions.^(5)^ However, there is a lack of clinical evidence to support this behavior. In two laboratory studies, ES during the procedure has been observed to increase the amount of secretions passing into the distal airway.^(5,8)^

Only 12.5% use PP during extubation. These results are consistent with those of another similar survey in which only 15% reported performing extubation with PP.^(6)^ It has been shown that, by applying PP, the release of airflow from the ventilator during deflation and removal of the ETT minimizes the aspirated volume.^(5,8)^ It is noteworthy that, despite knowing about the use of PP during extubation, more than half of the respondents do not use it. This result could be due to the fact that traditional maneuvers are not associated with relevant clinical complications, and in addition, there is little clinical evidence to support a change in the usual practice of extubation.^(10-12)^

According to the respondents in our study, the most common complication observed during extubation was stridor. This result coincides with that of a survey conducted among anesthetists in which it was also the most prevalent complication (35%), although its prevalence was lower than that observed in our sample.^(9)^ This difference could be due to the heterogeneity of the populations studied (for example, shorter duration of ETT use).

The high stridor rate reported by our participants (53%) should be noted. Because the respondents' answers refer to what they perceive, we consider this rate overestimated due to the role and clinical relevance of this complication in extubation failure.

The rate of desaturation (15%) reported in our study was similar to the rate of 22% reported by Rassam et al.^(9)^ As desaturation is one of the most commonly reported complications of orotracheal extubation (OTE) and is related to the loss of volume and oxygen stores that occur during ES, it has been examined in studies comparing different extubation techniques.^(10-12)^ Nevertheless, only one-third of our respondents reported increasing the oxygen supply before extubation.

This study has limitations. Among them is the low response rate of 44%. One explanation for this low response rate is that we did not know the number of inactive email accounts. The response rate was higher than the 28% reported in a similar study and higher than the average response rate in studies with online surveys (37%).^(6,13)^ This favorable difference could be due to the fact our survey was simple and brief to minimize ambiguity and lack of adherence. Most surveys were successfully completed.

We believe our respondents represent a broad spectrum of practice in the entire field studied. However, most professionals who answered the survey work in adult care, making it difficult to extrapolate the results to the pediatric population.

It is worth mentioning the potential risk of recall bias due to the retrospective nature of the variable "complications observed in the last three months." Therefore, our results should be interpreted with caution.

Finally, although a lower frequency of complications can lead to less extubation failure and subsequent reintubation, it is important to consider that these are related not only to aspects of the ETT removal procedure but also to patient factors related to spontaneous ventilation through the natural airways.

We can highlight that this is the first study in our field to report the procedures commonly used during patient extubation by critical care doctors and physical therapists with extensive experience and training. Although extubation is a common procedure, it has not been well studied, defined, or standardized.

## CONCLUSION

Most critical care professionals in the Autonomous City of Buenos Aires and in the Province of Buenos Aires, Argentina, use endotracheal suctioning without applying positive pressure during cuff deflation and extubation. The results of the present study justify conducting clinical studies to determine the effectiveness of and complications associated with the different maneuvers available to professionals at the time of extubation.
